# Molecular Effects of Biogenic Zinc Nanoparticles on the Growth and Development of *Brassica napus* L. Revealed by Proteomics and Transcriptomics

**DOI:** 10.3389/fpls.2022.798751

**Published:** 2022-04-25

**Authors:** Sohail, Laraib Sawati, Elenora Ferrari, York-Dieter Stierhof, Birgit Kemmerling, Zia-ur-Rehman Mashwani

**Affiliations:** ^1^Department of Botany, Pir Mehr Ali Shah (PMAS)-Arid Agriculture University, Rawalpindi, Pakistan; ^2^Center for Plant Molecular Biology (ZMBP), University of Tübingen, Tübingen, Germany; ^3^Institute of Biology/Plant Physiology, Humboldt-University Zü Berlin, Berlin, Germany; ^4^Department of Chemical and Life Sciences, Qurtuba University of Science and Information Technology, Peshawar, Pakistan

**Keywords:** nanotechnology, proteomics, *Brassica napus*, zinc nano fertilizer, green synthesis

## Abstract

Plants are indispensable on earth and their improvement in terms of food security is a need of time. The current study has been designed to investigate how biogenic zinc nanoparticles (Zn NPs) can improve the growth and development of *Brassica napus* L. In this study, Zn NPs were synthesized utilizing *Mentha arvensis* aqueous extracts, and their morphological and optical properties were assessed using UV-Visible spectrophotometry, scanning electron microscopy (SEM), transmission electron microscopy (TEM), and X-ray diffraction (XRD). The synthesized Zn NPs were irregular in shape, indicating aggregation in pattern, with an average particle size of 30 nm, while XRD analysis revealed the crystalline structure of nanoparticles. The growth and development of *B. napus* varieties (Faisal canola and Shiralee) were assessed after foliar treatments with different concentrations of biogenic Zn NPs. In *B. napus* varieties, exposure to 15 mg/L Zn NPs dramatically increased chlorophyll, carotenoid content, and biomass accumulation. Similarly, proteomic analyses, on the other hand, revealed that proteins associated with photosynthesis, transport, glycolysis, and stress response in both *Brassica* varieties were substantially altered. Such exposure to Zn NPs, differential expression of genes associated with photosynthesis, ribosome structural constituents, and oxidative stress response were considerably upregulated in *B. napus* var. (Faisal and Shiralee canola). The results of this study revealed that foliar applications of biogenic Zn NPs influence the transcriptome and protein profiling positively, therefore stimulating plant growth and development.

## Introduction

Plants extracts have the extraordinary potential to transform bulk metals into nanoparticles (NPs). They can reduce bulk material in an eco-friendly reaction into nanostructures. The photometabolites along with the reducing capabilities stabilize and provide surface functionalization attributes to the NPs (Javed and Mashwani, 2020b). The physical and chemical methods of reduction of bulk material involve the utilization of hazardous chemicals, which make nanostructures unsuitable for use in biological applications due to their toxic nature and harmful effects on plants, the environment, and other living organisms (Ahmad et al., 2020; Javed and Mashwani, 2020a). However, biofabrication using plant extracts results in the synthesis of biocompatible nanomaterials. Plants have the natural mechanism to absorb essential nutrients from the soil and reduce them into smaller particles, which latter assimilate and help improve growth and development of the plants (Javed et al., 2020).

Recent advances in nanotechnology allowed the development of NPs that can be widely used in an increasing number of different applications (Allhoff et al., 2010). Nanoparticles are aggregates of atoms or molecules with small size, usually less than 100 nm, and large surface areas, which drastically alter their physicochemical characteristics as compared with bulk materials (Love et al., 2005). The exposure of some plants to some NPs gives rise to reactive oxygen species (ROS) production, which has both positive and negative effects (Mazumder et al., 2020). Moreover, the nature, size, surface area, and composition of metal materials greatly influence the activity of NPs (Jain et al., 2007). The massive production and utilization of NPs are becoming a serious issue regarding their environmental impact (Klaine et al., 2008; Raghib et al., 2020). Plants are vital elements of the ecosystem and can react to NP exposition (Khot et al., 2012; Prasad et al., 2014). Therefore, the release of NPs into the environment, contact of NPs with plants, and their effect on plants, ecosystems, and the entire environment need a serious reconsideration.

Advanced nanotechnology has provided the technological platform to study the effects as well as mechanism of NPs effects in plants (Sahoo et al., 2007; Newman et al., 2009). The unique characteristics of nanosized particles (1–100 nm) have arouse the attention of diverse scientific communities, such as agronomy, technology, biology, and chemistry, because of their unique characteristics in comparison with bulk material (Ejaz et al., 2018). The NPs, which are made up of metal oxides, are used extensively for commercial purposes, such as antimicrobial soaps, topical sunscreens, and coatings for self-cleaning (Javed et al., 2020). The contamination of ecosystems by NPs is becoming a major concern due to their overproduction for use in daily consumables (Liu et al., 2021). The rapid development of the nanomaterial industry and the utilization of nanomaterial in mass products have caused difficulties in controlling the release of engineered NPs into the environment (Piccinno et al., 2012). However, there is very limited knowledge available about NPs effects on the environment and further studies are needed.

Zinc nanoparticles (Zn NPs) are widely used in many fields, e.g., agriculture and medicine. It was observed that Zn NPs can greatly influenced plant growth, yield, and fatty acid profiles of maize (Taheri et al., 2016). An increase in germination percentage and root length of maize plants was observed after exposure to zinc oxide nanoparticles (ZnO NPs) (Meena et al., 2017), whereas increased shoot length was observed in oat and berseem plants (Maity et al., 2018). It was also confirmed that ZnO NPs enhance the plant growth and increased root biomass of the *Glycine max* plant (De la Rosa et al., 2013). Foliar application of Zn NPs significantly increases the leaf area and dry mass of maize (Taheri et al., 2016). However, ZnO NPs and aluminum oxide NPs prevented root elongation of plants like cucumber, cabbage, corn, soybean, and carrot. The submergence effect on plants can be reduced by improving the duration of thresholds and flooding depth. Hossain et al. (2012) proclaimed that metallic NPs can protect plants from the detrimental effects of flooding by enhancing ATP generation rate and regulating the pathways of secondary metabolism. Zinc oxide nanoparticles can interact with plant roots in a various way. They can release Zn ions (Zn^2+^) that can be taken up by Zn transporters (Milner et al., 2013). Uptake might also happen through pores that are larger than the ZnO NPs (Fleischer et al., 1999). The rate of uptake and effectiveness of several NPs on growth and metabolism differ among various plants (Singh et al., 2018; Sturikova et al., 2018).

Keeping in mind the importance of zinc as a micronutrient, we present the environmentally friendly bioassisted synthesis of Zn NPs by using aqueous extracts of *Mentha* as an efficient oxidizing/reducing and capping agent. Although the biosynthesis of Zn NPs has been documented as *Brassica oleracea* (Awan et al., 2021), *Brassica juncea* (Mazumder et al., 2020), and *Brassica nigra* (Zafar et al., 2016), their varied effect on plant physiological and biochemical profiling has received less attention. The purpose of this study was to disclose the biological consequence of green synthesized Zn NPs on *Brassica napus* proteomics profiling, which was ignored in many published studies on *Brassica* crop (Mousavi Kouhi et al., 2015; El-Badri et al., 2021). In addition, different physiological and growth attributes have been investigated to strengthen this research. The idea was that Zn NPs would be advantageous at low doses, because higher dose may cause reduction in growth and lead to toxicity in plants. Plants are the foundational elements of the ecosystem, providing the initial energy for the food chain’s major consumers; therefore, *Brassica* crop has been used as model plant in this study.

## Materials and Methods

### Plant Extract Preparation

The aqueous leaf extracts of *Mentha arvensis* were used as a reducing and capping agent to produce Zn NPs. Fresh and healthy leaves were vigorously washed with tap water and distilled water. Notably, 20 g of leaf material were mixed with 100 ml of Milli-Q^®^ water and incubated at 50°C for 4 h. The extract was filtered two times by using Whatman filter paper No. 21 and kept at 4°C for further use (Dobrucka and Długaszewska, 2016).

### Green Synthesis of Nanoparticles

The green synthesis of NPs was performed by the reduction of ZnNO_3_⋅7H_2_O (zinc nitrate heptahydrate) using plant aqueous extract. In this process, 5-mM solution of Zn salt was prepared using Milli-Q^®^ water and was kept on a magnetic stirrer followed by the continuous addition of 100 ml of *Mentha* plant aqueous extract dropwise for 40 min. The color of the reaction mixture was changed to light brown, indicating the synthesis of Zn NPs. However, the synthesis of Zn NPs was confirmed by measuring the absorbance of the colloidal suspension of NPs between 200 and 800 nm of the light wavelength using a UV-visible spectrophotometer (Beckman Model DU640). This reaction solution was centrifuged at 1,000 × *g* for 1 h. The Zn NPs pellet was collected carefully, followed by dispersion in ethanol and re-centrifugation. The procedure was repeated two times to remove impurities. The Zn NPs were placed in the oven at 100°C to completely dry. The dried Zn NPs were stored in airtight vials for future use.

### Morphological and Optical Characterization

The biosynthesized Zn NPs were subjected to different material characterization techniques in order to determine their morphological, optical, and biochemical attributes (Almessiere et al., 2020). The scanning electron microscopy (SEM) SIGMA model (MIRA3; TESCAN Brno) and transmission electron microscopy (TEM, JEM-2200FS) were used to collect the micrographic images (Sohail et al., 2019). The drop-coating method was used to prepare the samples, and the images were collected at different voltages and laser intensities. The crystalline nature of Zn NPs was confirmed by using the X-ray diffraction (XRD, Bruker D2 Phaser) analysis with the monochromatic Cu-K_α1_ radiation at a 2θ angle between 10° and 80° (Sohail et al., 2020).

### Plant Materials and Growth Conditions

Locally available seeds of *B. napus* varieties (e.g., Faisal canola and Shiralee) collected from the National Agricultural Research Center, Islamabad, Pakistan, were grown directly in 5.5 cm × 5.5 cm pots with TS-1 white peat bedding substrate and were kept in dark for 2 days at 4°C. Later, the plant pots were transferred to a climate chamber at a temperature of 22°C with light intensity (photosynthetic active radiation) of approximately 120 mol/m^2^s and a light/dark photoperiod of 16 h/8 h.

### Zinc Nanoparticles Treatment

For proteomics analysis, seeds of canola varieties were sown in pots, and two consecutive sprays of Zn NPs (i.e., 5, 15, and 25 mg/L) were made after seedling emergence. One-week-old plants were treated with 5, 15, and 25 mg/L of biosynthesized Zn NPs by foliar spraying, followed by a second spray at day 3 after the first treatment.

### Biomass and Water Content Measurement

Four-week-old plants from each pot of three biological replicates were harvested and washed thoroughly with tap water and distilled water. The moisture was removed by using a paper towel. The roots and shoots were separated, and fresh weight (FW) was measured. The sample was then dried in an oven at 80°C for 20 min, followed by vacuum dry at 40°C to constant mass before recording dry weight (DW). The plant water contents were calculated using the following formula (Whetherley, 1950):


$$W\,a\,t\,e\,r\,C\,o\,n\,t\,e\,n\,t=\frac{F\,W-D\,W}{T\,D\,W}$$

### Chlorophyll and Carotenoid Content Measurement

To determine the chlorophyll and carotenoid contents, rosette leaves of treated plants were harvested and transported in polythene zipper bags. The leaves were washed with tap and distilled water and homogenized with 80% acetone followed by centrifugation for 10 min at 14,000 rpm. The supernatant was collected and subjected to UV-visible spectrophotometry analysis. The chlorophyll a (Chl a), chlorophyll b (Chl b), total chlorophyll (Chl T), and carotenoid (Car) contents were measured using the methodology described by Arnon (1949). The chlorophyll content was determined using methodology described by Lichtenthaler and Wellburn (1983).

### Protein Extraction

For the preparation of GTEN buffer, the buffer was mixed in the distilled water and kept at 4°C. The extraction buffer was prepared in a prechilled tube by equipping GTEN buffer with 10 mM dithiothreitol (DTT), 0.2% Nonidet-40 (Igepal), antiprotease tablet (Sigma-Aldrich) (1 tablet per 10 ml), and 2% polyvinylpyrrolidone (PVPP). About 100 mg of fresh leaves were powdered in liquid nitrogen using a pestle and mortar. The powder was mixed with 150 mM GTEN buffer (10% glycerol, Tris–HCl, pH 7.5), 1 mM ethylenediaminetetraacetic acid (EDTA), 150 mM NaCl, 10 mM DTT, 0.2% Nonidet-40 (Igepal), antiprotease tablet complete*™* Proteasehemmer-Cocktail (1 tablet for 10 ml), and 2% PVPP. The thawed material was incubated on a rotator for 20 min at 4°C. Each sample was then centrifuged at 5,000 × *g* at 4°C for 20 min to remove cell debris. The supernatant was collected carefully and shifted to a new prechilled tube on ice. A second centrifugation at 5,000 × *g* at 4°C for 5 min of each sample was carried out and the collected supernatant was shifted to a prechilled tube by filtering through Miracloth. Total protein concentration in the sample was investigated by using Bradford’s (1976) assays.

### Digestion of Protein for Mass Spectrometry Analysis

For proteomics analysis, proteins were obtained from Center of Proteome, University of Tübingen, Tübingen, Germany. The proteins were separated on a short SDS page and visualized by Coomassie staining. Gel pieces were in-gel digested with trypsin (Borchert et al., 2010). The extracted peptides were desalted and then labeled using C18 Stage Tips (Rappsilber et al., 2007; Boersema et al., 2009). Dimethyl labeling is a technique that used a specific reagent (i.e., cyanoborohydride and formaldehyde in unlabeled and stable isotope-labeled forms) in order to tag the primary amine, i.e., N-terminus and the ε-amino group of lysine in peptides, in proteins (Yan et al., 2020). Finally, the samples were labeled with dimethyl “light” (CH_3_)_2_ and dimethyl “intermediate” (CH_1_D_2_)_2_. Saturating incorporation levels of the dimethyl labels were achieved in all cases.

### Nano-Liquid Chromatography-Tandem Mass Spectrometry Analysis

Nano-liquid chromatography-tandem MS analysis was carried out, and eluted peptides were mixed in a 1:1 ratio conferring to measured protein amount. The analysis of the peptide mixture was achieved on an Easy-nLC 1200 System (Thermo Fisher Scientific) coupled with LTQ Orbit Rap Elite MS (Thermo Fisher Scientific). The peptides were separated with solvent A (0.1% of formic acid) at a flow rate of 500 nl/min and subsequently eluted with a 230 min gradient of (10-33-50-90%) HPLC solvent B (80% acetonitrile in 0.1% of formic acid) with a constant flow rate of 200 nl/min. The 15 most intense precursor ions were sequentially fragmented in each scan cycle (Boersema et al., 2009).

### Protein Identification From the Mass Spectrometry Data

The MS data were processed by using MaxQuant software suite version 1.5.2.8 (Cox and Mann, 2008), while database search was performed by using the Andromeda search engine (Cox et al., 2011), which is a module of the MaxQuant software. The MS/MS spectra were searched against protein entries from *B. napus*, and a database consisting of 285 commonly observed contaminants and reverse decoy matches were removed from the protein identification list. In a database search, full tryptic specificity was required and up to two missed cleavages were allowed. The protein N-terminal acetylation and oxidation of methionine were set as variable modifications. The peptide, protein, and modification-site identifications were filtered using a target-decoy approach at a false discovery rate (FDR) set to 0.01 (Elias and Gygi, 2007). A minimum of two quantified peptides were registered for protein group quantification. The Perseus software (version 1.5.0.15), a module from the MaxQuant suite (Tyanova et al., 2016), was used for the calculation of the significance *B* (*p* sig. *B*) for each protein ratio with respect to the distance of the median of the distribution of all protein ratios as well as its intensities. All proteins with *p* sig. *B* < 0.1 in a pairwise comparison were considered differentially expressed.

### RNA Extraction

For total RNA extraction (100 mg), leaf tissue was powdered in liquid nitrogen using mortar and pestle. Total RNA extraction was carried out by using MACHEREY-NAGEL KIT Germany and treated with RNase-free DNase I during extraction. The concentration was checked using NanoDrop.

### Reverse Transcription/cDNA Synthesis

To synthesized cDNA from the extracted RNA, synthesis was carried out using the protocol of Cello et al. (2002). The extracted RNA was equipped with 2 μl of oligo dT primer 0.5 mM and kept incubated for 10 min at 70°C followed by incubation for 2 min on ice. Precise amount of 8 μl of Reveres Transcription (RT) Buffer, 4 μl of dNTP, 2 μl of Reverse aid RT (company), and 1 μl of Ribo lock (Thermo Fisher Scientific) was added to the reaction mixture. The mixture was then incubated for 90 min at 42°C. The incubated mixture (cDNA) was stored at −20°C for future use (Table 1).

**TABLE 1:** Details of selected genes and primers designed by using the NCBI primer-BLAST tool for the quantitative real-time PCR (qRT-PCR) analysis of the plants treated with 15 mg/L of Zn NPs.

Plants	Gene/Accession No	5′ Primer	3′ Primer
Faisal canola	BnaC09g00220D/PsaD	TGCTTGAATTC CTAAGTTTGC	AATATGCCCA TTCCCATCAA
	BnaC03g44500D	ATTGTCAAGG CTTCTGCTTA	AGAATGAAAT GCTCTCACCT
	BnaA01g14450D/FSD1	GGTCCACTAA GGAAGAAACA	TCCTAGCTTCG GCTATATCA
Shiralee	BnaC09g00220D/PsaD	TTGTCGTTG TCGTTAAAACC	TTGCTTAAAC CATGCTACGA
	BnaA04g15980D	GAGTTGATTG CTGTTGGAAG	CCCATGTCCT GCAAATTAAC
	BnaAnng33440D	TTGGTTTGTC CATGTTGTGA	CCGAGAATGAG TAACGAGTA

*Three biological replicates were used for gene expression.*

### Quantitative Real-Time PCR (qRT-PCR) for Gene Amplification

Real-time quantitative PCR analysis was performed with Bio-Rad iQ5 Thermo Fisher. The reaction mixture was prepared by mixing SYBR^®^ Green dye, 10 μM of forward primer and reverse primer, and 1.5 μl of RNA-free water. Later, 1 μl of cDNA of *B. napus* cultivars were added to each well. All steps were performed essentially according to the manufacturer’s protocol. Relative gene expression calculation was performed with the Bio-Rad CFX-Manager Software version 1.6 using the DDC(t)method and ACTIN as reference.

### Statistical Analysis

All the experiments were performed in triplicates using three different biological replicates. The data were analyzed statically using SPSS version 20 software for the analysis of variance (ANOVA) and the mean significant differences were separated using Duncan’s multiple range test (DMRT).

### Availability of Data and Materials

The proteome data set of this study is available as supplementary material. The biogenic Zn NPs and plants material are available from the corresponding authors on reasonable request.

### Ethics Statement

This material is the authors’ own original work, which has not been previously published elsewhere. This study does not involve any human or animal trials and was purely based on the use of green synthesized Zn NPs for the growth and development of rapeseed plant.

## Results and Discussion

### Morphological and Optical Characterization of the Photosynthesized Zinc Nanoparticles

**FIGURE 1:**
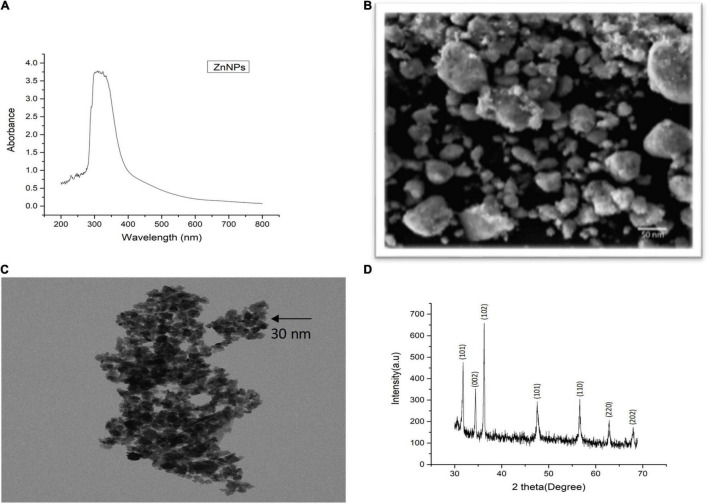
Characterization of green synthesized Zn NPs using *Mentha arvensis* leaves with **(A)** UV-visible spectroscopy, **(B)** scanning electron microscopy (SEM), **(C)** transmission electron microscopy (TEM), and **(D)** X-ray diffraction analysis (XRD). SEM analysis showed aggregated nature of Zn NPs in the solution used, indicating a gel-like appurtenance, while clear morphology of the green synthesized NPs can be seen in TEM image, revealing an irregular shape. The XRD peaks are in reference to the standard present in the library data of the XRD machine, which confirmed the presence of Zn NPs in the sample.

Plant-mediated green synthesis is considered an eco-friendly approach for the synthesis of biocompatible NPs. In this study, Zn NPs were synthesized by using *M. arvensis* aqueous extract as a reducing and stabilizing agent (Sohail et al., 2019). A surface plasmon resonance (SPR) band characteristic of Zn NPs was observed at 292 nm wavelength (Figure 1A). The SPR band is a response of the interaction of the oscillating electromagnetic light waves with the NPs resonance (Yasmeen et al., 2017). The SEM images visualize mostly the surface of the Zn NPs. Most of the Zn NPs are visible as aggregates (Figure 1B). The TEM images revealed that most of the NPs exist in the size range of 30–50 nm. The morphological evaluation of the Zn NPs photosynthesized by *M. arvensis* exhibits an irregular shape and a size of the NPs that is suitable for plant exposition experiments (Figure 1C). The NPs appear aggregated, confirming the SEM analysis results obtained for these NPs. Transmission electron microscopy reveals the irregular shape of Zn NPs with a similar size range as revealed by SEM. The XRD analysis confirmed the crystalline nature of the Zn NPs, while different peaks represent the pattern and phase angle of the synthesized NPs (Figure 1D). The diffraction peaks were observed at the angle of 14.3°, 17.6°, 27.6°, 47.8°, 51.6°, 65.7°, 73.4°, and 80.2° with their corresponding lattice plane. These angels are analogous to (101), (104), (002), (110), (111), (220), and (202) Miller indices, respectively. The XRD analysis exhibits sharp peaks that are referenced according to the Joint Committee Powdered Diffraction (JCPD) number, proving the presence of Zn NPs in the sample (Lu and Yeh, 2000).

### Chlorophyll and Carotenoid Content of the *Brassica napus* Varieties Treated With Zinc Nanoparticles

To investigate the effects of these Zn NPs on the physiological responses of the canola varieties, i.e., Faisal and Shiralee, the 4-week-old plants were treated with 5, 15, and 25 mg/L of Zn NPs, while untreated plants were considered controls. Chlorophyll and carotenoids play an indispensable role in the photosynthetic process largely determining the photosynthetic capacity of the plants. Chlorophyll and carotenoid contents can alter depending on the growth conditions, health status, and other environmental factors (Pinto et al., 2020).

**FIGURE 2:**
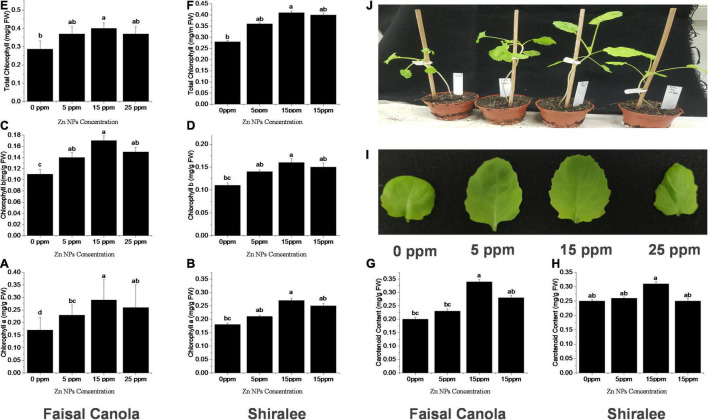
Effects of Zn NPs on chlorophylls and carotenoids contents in *Brassica napus* rosette leaves. **(A,B)** Chlorophyll a, **(C,D)** Chlorophyll b, **(E,F)** total chlorophylls, and **(G,H)** carotenoids in rosette leaves of 5-week-old *B. napus* (Faisal canola and Shiralee), **(I)** leaf size, and **(J)** phenotype of plants treated with 0, 5, 15, and 25 ppm of Zn NPs. Chlorophylls and carotenoids were extracted from rosette leaves of 5-week-old *B. napus* plants using 80% acetone and measured using a spectrophotometer. Data represent the mean ± SD of three replicates. Different letters indicate significantly different (*p* < 0.05).

Zinc nanoparticles treatment had a positive effect on chlorophyll contents in both Faisal and Shiralee *B. napus* varieties. Plants treated with Zn NPs had a significantly increased level of Chl a, Chl b, and Chl T content after Zn NPs treatment with all concentrations applied. The highest increase was obtained with 15 mg/L Zn NPs. The Chl a content was enhanced by 47 and 50% in both canola varieties, respectively, after foliar application of 15 mg/L of Zn NPs (Figures 2A,B). The Chl b content was 54 and 46% higher in both canola varieties, respectively, with this treatment (Figures 2C,D). The Chl T content with 15 mg/L of Zn NPs application showed 46% increase in Shiralee and 50% increase in Faisal (Figures 2E,F).

The carotenoid content was increased by 65% in Faisal canola and 34% in Shiralee with 15 mg/L of Zn NPs (Figures 2G,H). There has been a considerable rise in leaf size on the same treatment, and the phenotypic also correlates with this effect of Zn NPs (Figures 2I,J).

The effects of different concentrations of ZnO-ZnO-NPs on the photosynthetic pigments (i.e., chlorophyll and carotenoids) were investigated in peanut (Prasad et al., 2012), pearl millet (Tarafdar et al., 2014), green pea (Mukherjee et al., 2014), cluster bean (Raliya and Tarafdar, 2013), and tomato (Raliya et al., 2015) and found a positive effect. These studies support our results in that Zn NPs are growth promoting and do not cause stress or damage to plants. Zinc plays an important role in the synthesis of chlorophyll, DNA replication, nitrogen fixation, and electron transport chain (e.g., chloroplast and mitochondria) (Yruela, 2005; Nouet et al., 2011), indicating that it is essential for plant health and that supplementing with Zn may have growth-promoting effects. It also plays a protective role in photo-oxidative stress; for example, plants growing in zinc-deficient environments are more vulnerable to drought-induced oxidative stress (Cakmak, 2000). Marusenko et al. (2013) have explained experimentally that ZnSO_4_ (salt) showed a smaller increase in chlorophyll content compared with plants treated with ZnO NPs, suggesting that the NPs can offer advantages over traditional bulk Zn applications (Faizan et al., 2021).

### Zinc Nanoparticles Influence the Growth and Biomass Accumulation in the *Brassica napus* Varieties

To evaluate the effect of NPs on plant performance, morphological characteristics such as shoot FW and DW were measured.

**FIGURE 3:**
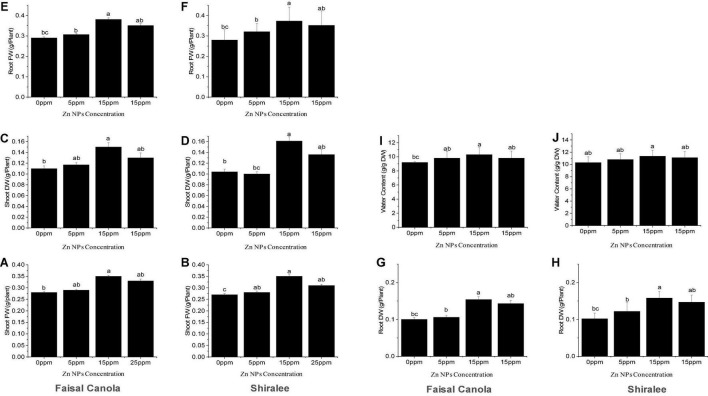
Effect of zinc nanoparticle significantly influences biomass accumulation shoot fresh **(A,B)** and dry weight **(C,D)**, root fresh **(E,F)** and dry weight **(G,H)**, and relative water content **(I,J)** in *Brassica napus* varieties, i.e., Faisal canola and Shiralee. Data were expressed in mean ± SD. Bar with different letters indicates significance, while bar with similar letters indicates non-significance. Three different biological replicates were used in the study.

After exposure to Zn NPs, the FW and DW of the shoots increased significantly in the rapeseed varieties. The shoot FW was significantly higher at 15 mg/L of Zn NPs, with an increase in 25% in Faisal canola and 29% in Shiralee as compared with the untreated control plants (Figures 3A,B).

In contrast, the Zn NPs showed a slight increase in the FW of the shoot after treatment with 5 mg/L, with 3.5% in Faisal canola and 4.7% in Shiralee.

None of the concentrations tested resulted in decreased biomass, showing that Zn NP treatment enhanced growth and has no detrimental but positive effects on canola FW. This means that the canola variety Faisal responds more strongly to the treatment than do Shiralee. The shoot DW was significantly higher at 15 mg/L of Zn NPs, with an increase in 36% in Faisal canola and 51% in the Shiralee variety, indicating that the water content of Faisal contributed stronger to the FW gain than do Shiralee that shows higher DW and therefore higher biomass increases (Figures 3C,D). Generally, Zn NPs application significantly improved the FW and DW in both the study canola varieties.

The roots of the canola plants showed a similar effect upon treatment with 15 mg/L of Zn NPs. The root FW showed an increase by 31% for Faisal canola and 33% for Shiralee as compared with control plants (Figures 3E,F). In the case of Zn NPs, a dose-dependent tendency was observed at different concentrations, i.e., 5 mg/L of Zn NPs caused an increase by 5% in Faisal canola and 14% in Shiralee canola whereas 25 mg/L of Zn NPs caused 10% in Faisal and 20% in Shiralee (Figures 3G,H). The root DW was increased by 54% in Faisal canola and 58% in Shiralee canola at 15 mg/L of Zn NPs. Therefore, the variety Shiralee responded significantly higher than Faisal canola.

Positive effects of Zn NPs on plant growth have been reported previously in other plant species, supporting our findings. Tarafdar et al. (2014) reported improved wheat plant height, root length, and root weight with the foliar application of Zn NPs. Similarly, an increase in the root/shoot biomass of *Vigna radiata* was observed (Dhoke et al., 2013; Taheri et al., 2016). Enhanced root/shoot DW was reported in the sunflower by ZnO NPs exposure (Torabian et al., 2016). ZnO NPs promote onion growth and reduce the flowering period at 20 and 30 mg/L concentrations (Laware and Raskar, 2014). Burman et al. (2013) reported an improved growth in the chickpea with foliar application of Zn, whereas Prasad et al. (2012) reported increased root/shoot growth in peanut plant.

### Plant Relative Water Content Measurement in Response to Zinc Nanoparticles

The relative water content (RWC) acts as a valuable physiological indicator to plants. The foliar application of 15 mg/L of Zn NPs significantly increased the RWC of both canola varieties (i.e., 11% for the Faisal canola and 10% for the Shiralee) (Figures 3I,J). A slight increase in the RWC was observed in relation to the seeds treated with 5 mg/L of Zn NPs, which showed an increase in 6% in Faisal canola and 4% in Shiralee. These findings resulted that the RWC of both canola varieties showed a significant increase after Zn NPs applications. Many researchers have indicated the importance of Zn in plant growth, development, reproduction, and yield (Camp and Fudge, 1945; Chapman, 1966; Anderson, 1975; Brown et al., 1993; Marschner, 1993; Stampoulis et al., 2009). Khan et al. (1998) reported that Zn can improve the water content of plants. They observed that the addition of Zn increased the water content of leaf and stomatal conductance in chickpea plants. Deficiency of Zn leads to water stress and reduced stomatal conductance and the water-use efficiency of plants (Khan et al., 2003). Thus, as Zn NPs can improve the photosynthetic pigment content, growth, and water content of plants, they should be used to improve the quality of rapeseed and other crop plants.

### Effect of Zinc Nanoparticles on Proteomic Profiling of *Brassica napus*

**FIGURE 4:**
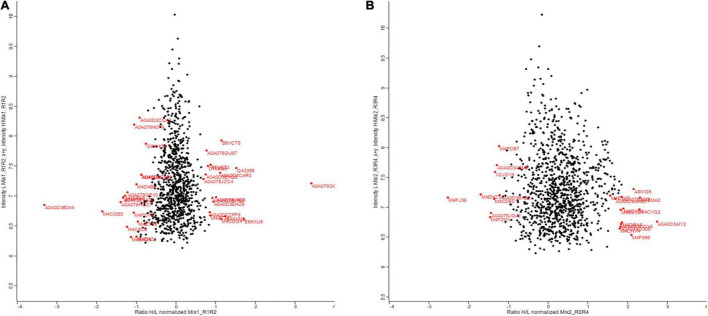
Summary of the results comparing deep-scale proteome analysis data of *Brassica napus* variety **(A)** Faisal canola and **(B)** Shiralee using nano-flow LC-MS/MS data and data obtained by micro-flow LC-MS/MS data in this study. Red color indicates significantly altered proteins. The red on the right side of the scale starting from 0 shows an increase in protein expression while the red on the left side of 0 indicates the decrease in protein expression. Three different biological replicates were used in the study.

Zinc nanoparticles have a positive effect on plant biomass. To understand how this is achieved, we performed proteomic analysis. Also, to find out which proteins are changed as a result of Zn NP treatment and whether this can explain why the plants perform better. Proteins were extracted from the leaves of a 30-days-old canola plant treated with 15 mg/L of Zn NPs and analyzed by gel-free/label proteomics technique. Both canola varieties showed a significant alteration in their protein profiles. A total of 1,904 proteins were identified from protein extracts by nano-LC/MS-MS, with 35 of the proteins from Faisal canola and 23 proteins from Shiralee being significantly altered (Figures 4A,B).

Proteomic profiling of the *B. napus* variety Faisal canola revealed that various proteins involved in the photosynthesis, carotenoid biosynthetic pathways, light-harvesting protein-chromophore linkage, signal transduction, glutamine metabolic process, cysteine biosynthetic process from serine, heme-binding proteins, iron ion-binding proteins, defense-related protein, NAD(P)H dehydrogenase (quinone) activity, and water-soluble chlorophyll protein (WSCP) were significantly altered in zinc-treated plants (Table 2). Metallic NPs such as the ZnO NPs mainly target the cellular organization mechanism and hormonal and protein-related secondary metabolism under overflowing stress (Hossain et al., 2012). In contrast, *B. napus* variety Shiralee protein related to photosynthesis, protein metabolic process, defense response, glycolytic process, transport, carbohydrate metabolic process, translation, defense response to biotic stimulus, and carbon fixation was altered in plants treated with Zn NPs (Table 3).

**TABLE 2:** Quantitative proteomics table of proteins with differential abundance (ratio below 0.8 or above 3.00 relative to the Zn NPs-treated plants).

Protein IDs	Species	GOMF name	GOBP name	Gene name	Ratio H/L normalized
M4CGE0	*B. rapa*	Hydrolase activity, hydrolyzing o-glycosyl compounds	Carbohydrate metabolic process	Glu1	−1.857835
E5KXU6	*B. campestris*	Copper ion binding; metal ion binding; protein domain specific binding; superoxide dismutase activity	Circadian rhythm; response to cadmium ion; response to copper ion; response to light intensity; response to ozone	FSD1	1.709247
A0A0D3C833	*B. oleracea v*	ATP binding; metallo-endopeptidase activity; zinc ion binding		106292516	−0.8753542
A0A0D3BYR6	*B. oleracea*	Oxidoreductase activity, acting on paired donors, with incorporation or reduction of molecular oxygen; oxidoreductase activity	Carotenoid biosynthetic process	PDS	0.9167823
M4FDA0	*B. rapa*	Chlorophyll binding	Photosynthesis		−0.8749309
Q9XHG8	*B. napus*	Channel activity	Transport	gamma-TIP2	−0.7562578
M4FD78	*B. rapa*	Proton transmembrane transporter activity	Transport		−1.066609
A0A078G6S6	*B. napus*	ADP binding	Signal transduction	BnaA01g33720D	3.414677
A0A078GAD3	*B. napus*	Cysteine synthase activity	Cysteine biosynthetic process from serine	BnaC04g49280D	−1.329051
M4EZJ4	*B. rapa*	Heme binding; iron ion binding; monooxygenase activity; oxidoreductase activity, acting on paired donors, with incorporation or reduction of molecular oxygen		BnaAnng14260D	0.8665519
A0A078GE10	*B. napus*	Carbohydrate binding	Lectin Protein	BnaA06g01990D	−1.226274
Q93XM2	*B. carinata*	Defense related protein	Glutamine metabolic process	CJAS1	1.232845
A0A0D2ZPF8	*B. oleracea*	Uncharacterized protein	Uncharacterized protein	BnaC02g36330D	0.8458312
A0A078GU87	*B. napus*	Carbonate dehydratase activity; zinc ion binding	Carbon utilization	BnaCnng05580D	0.7713785
A0A078H0T9	*B. napus*	Cysteine synthase activity	Cysteine biosynthetic process from serine	BnaA04g25390D	−1.053224
A0A078HQZ7	*B. napus*	Hydrolase activity, hydrolyzing o-glycosyl compounds; xyloglucan: xyloglucans transferase activity	Cell wall biogenesis	BnaA03g50050D	−1.396688
M4CDG4	*B. rapa*	L-aspartate:2-oxoglutarate aminotransferase activity; pyridoxal phosphate binding	Biosynthetic process; cellular amino acid metabolic process	106320409	1.128755
M4CFK0	*B. rapa*	FMN binding; NAD(P)H dehydrogenase (quinone) activity	Oxidation-reduction process	BnaCnng38420D	−1.283878
M4EJQ9	*B. rapa*	FMN binding; NAD(P)H dehydrogenase (quinone) activity	Oxidation-reduction process	106331476	−1.138569
B6VCT5	*B. rapa*	Epithiospecifier protein	Post-translational modifications	BnaAnng10080D	1.142087
A0A078J0C6	*B. napus*	Electron transfer activity	Post-translational modifications	106305373	1.004106
Q7X737	*B. juncea*	Transferase activity	Detoxification	GSTF2	−1.337922
A0A078JZC4	*B. napus*	Hydrolase activity, hydrolyzing o-glycosyl compounds	Carbohydrate metabolic process	BnaA09g52790D	0.6890308
A0A0D3B244	*B. oleracea*	Manganese ion binding; nutrient reservoir activity		106329205	−3.31919
M4C8C6	*Brassica rapa*	Isomerase activity	Cell redox homeostasis	106328378	−1.231617
A0A0D3BXQ9	*Brassica oleracea*	Triose-phosphate isomerase activity	Glycolytic process; metabolic process	106342295	0.7464848
A0A0D3C4R0	*Brassica oleracea*	RNA binding	Uncharacterized protein	106342759	1.1143
A0A0D3CQ47	*Brassica oleracea*	Carbohydrate binding	Uncharacterized protein	106298242	−0.9183059
A0A0D3EHZ6	*Brassica oleracea*	Glutamate synthase activity	Glutamate biosynthetic process	106317861	0.9386246
D1L8Q3	*Brassica napus*	Photosystem ii reaction center protein l	Photosynthesis	psbL	0.8029789
B5U8Z3	*Brassica rapa*	Hydrolase activity, acting on carbon-nitrogen (but not peptide) bonds	Nitrogen compound metabolic process	BrNIT2	0.8696346
M4D4B0	*Brassica rapa*	ATP binding; l-aspartate:2-oxoglutarate aminotransferase activity; protein serine/threonine kinase activity; pyridoxal phosphate binding; transaminase activity	Cellular amino acid metabolic process;		−0.9932066
M4EHZ1	*Brassica rapa*	Monooxygenase activity; ribulose-bisphosphate carboxylase activity	Carbon fixation; photorespiration; photosynthesis		−0.9936661
M4ET89	*Brassica rapa*	Oxidoreductase activity	Oxidation-reduction process	BnaA05g32330D	−0.96508
Q43395	*Brassica napus*	Endopeptidase inhibitor activity	Uncharacterized protein	WSCP	1.518787

*GOMF, gene ontology molecular function; GOBP, gene ontology biological function.*

**TABLE 3:** Quantitative proteomics table of proteins with differential abundance (ratio below 0.8 or above 3.00 relative to the Zn NPs-treated plants).

Protein IDs	Species	GOMF name	GOBP name	Gene name	Ratio H/L normalized
M4EHZ1	*B. rapa*	carbon fixation; photorespiration	Photosynthesis		−1.70524
A0A0D3AHJ2	*B. oleracea*	Structural constituent of ribosome	Translation		1.688717
M4EYV7	*B. rapa*	Structural constituent of ribosome; ubiquitin-like modifier Activating enzyme activity	Translation	106300706	1.558219
A0A0D3A5M6	*B. oleracea*	Apoplast; vacuole	Cellular amino acid metabolic process	BnaA01g13280D/106313559	−1.29138
A0A078GI73	*B. napus*	Structural constituent of ribosome	Translation	BnaA06g12670D	1.842456
A0A078G305	*B. napus*	ND	Ribosome biogenesis	BnaA09g03590D	1.829159
M4F2M1	*B. rapa*	Allene oxide synthase activity; heme binding; heme binding; iron ion binding; monooxygenase activity; oxidoreductase activity, acting on paired donors, with incorporation or reduction of molecular oxygen	Defense response to fungus; jasmonic acid biosynthetic process; oxylipin metabolic process; response to jasmonic acid; response to wounding	AOS	−1.45815
M4EZX0	*B. rapa*	Structural constituent of ribosome	Translational elongation	BnaA04g15980D	1.821955
A0A0D3BXB6	*B. oleracea*	Glucan endo-1,3-beta-D-glucosidase activity; hydrolase activity, hydrolyzing O-glycosyl compounds	Carbohydrate metabolic process; defense response	BnaC04g24330D/106341843	−1.22014
X2JGY9	*B. rapa*	Calcium ion binding; calcium-dependent phospholipid binding		106295437	−1.34015
M4F066	*B. rapa*	Hydrolase activity, acting on ester bonds	Uncharacterized protein	106292567	2.09285
A0A078J0U0	*B. napus*	Serine-type endopeptidase activity	Hydrolase, Protease, Serine protease	BnaCnng28690D/106313216	−1.43511
M4EMA2	*B. rapa*	ATP binding	Protein metabolic process	106311155	2.309933
A0A0D3AIY2	*B. oleracea*	Integral component of membrane	Transport	106319205	2.740755
M4CNY4	*B. rapa*	Aspartic-type endopeptidase activity	Protein catabolic process	106328540	1.804962
A0A0D3CDX6	*B. oleracea*	DNA binding; DNA-directed 5′-3′ RNA polymerase activity; protein dimerization activity; structural constituent of ribosome	Transcription, DNA-templated; transcription, DNA-templated; translation	rpoA	1.832607
A8IXG5	*B. campestris*	Signaling receptor activity	Defense response; response to biotic stimulus	BnaAnng33440D	2.154389
M4CDE0	*B. rapa*	ATP binding; glucose-1-phosphate adenylyl transferase activity	Photosynthesis	106319363	−1.35657
M4CYK1	*B. rapa*	Aspartic-type endopeptidase activity	Protein catabolic process	106319255	1.89309
M4CYQ2	*B. rapa*	Uncharacterized protein	Uncharacterized protein	BnaC09g47440D	2.296604
M4DRA6	*B. rapa*	Photosynthesis, metal ion binding; protein domain specific binding; protochlorophyllide reductase activity	Photosynthesis	BnaA03g48610D	1.858459
M4ECB7	*B. rapa*	Fructose-bisphosphate aldolase activity	Glycolytic process		−1.23016
M4FJ36	*B. rapa*	Ribulose-bisphosphate carboxylase activity	Carbon fixation		−2.53191

Protein alterations in the plant after exposure to NPs could provide a novel biomarker by using advanced bioinformatics tools (Roy et al., 2017). Studies on green synthesized NPs affecting plant morphology and proteomics profiling are limited. Yasmeen et al. (2016) observed that aluminum oxide NPs significantly altered the number of proteins in the soybean plant, which leads to the discovery of a protein involved in signaling and protein metabolism process. Similarly, silver NPs were reported to altering the proteins involved in the cellular metabolism and stress signaling responses (Mustafa et al., 2015).

Our data on the differential expression of proteins provide a valuable data set that can be used to study specific physiological effects of Zn NPs on canola and may be also used to extrapolate on other plants.

### Differential Expression Analysis of Candidate Genes

**FIGURE 5:**
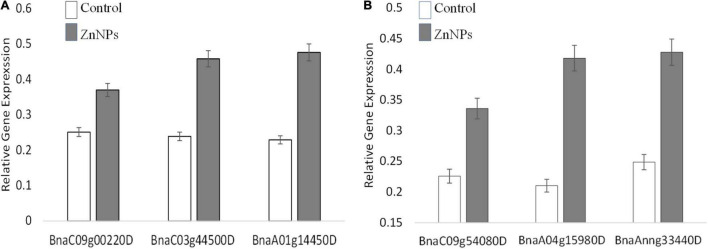
Expression of the randomly selected gene from *Brassica napus* varieties **(A)** Faisal canola and **(B)** Shiralee were assisted using quantitative real-time PCR Bio-Rad iQ5 Thermo Fisher. Four-week-old Zn NPs treated (15 mg/L) plants were harvested, RNA was extracted, and cDNA was synthesized. Represented values are the mean ± SD obtained from three individual experiments performed for each sample.

Proteomic analyses revealed numerous proteins were influenced differently after exposure to Zn NP. In this study, three genes in each variety were selected and their expressions were checked at the transcriptional level. The selection of genes was based on the most altered expression of their proteins in proteomic analyses. Actin (ACT7) was used as a reference gene, and three biological replicates were analyzed on the transcriptional level. The gene responsible for the photosynthesis, superoxide dismutase (SOD), and ribosome structural components (Protein ID/Gene: *BnaC09g00220D/PsaD*, *BnaA01g14450D/FSD1*, and *BnaC03g44500D*) in Faisal canola and (*BnaC09g00220D/PsaD*, *BnaAnng33440D*, and *BnaA04g15980D*) in Shiralee were significantly upregulated in canola varieties on exposure to Zn NPs at 15 mg/L as compared with control plants (Figure 5A). The gene associated with photosynthesis showed more expression/upregulation, followed by gene involved in SOD activity as compared with control plants (Figure 5B).

To date, only a few studies have been carried out to identify differentially expressed genes after Zn-NP exposure. Landa et al. (2012) reported differential expression upon Zn NPs exposure including a putative carbonic anhydrase family protein (At1g58180), Naringenin 3-dioxygenase (At3g51240), 2-oxoisovalerate dehydrogenase (At1g21400), and an acidic phosphatase class B family protein (At4g29270). Numerous genes were upregulated and downregulated in *Arabidopsis thaliana* upon exposure to Ag NPs (Xu et al., 2011). Due to very limited literature, the function of Zn NP in the gene expression is not very cleared.

## Conclusion

This study reports the effects of biosynthesized and therefore biocompatible and non-toxic Zn NPs on two different canola varieties. Multiple characterization results revealed that the Zn NPs are 30 nm in size and showed aggregation with irregular shape and morphology, which makes them suitable for various biological applications. All tested concentrations of the Zn NPs have positive effects on both canola varieties, with 15 mg/L giving the strongest effects. Chlorophyll and carotenoid content, growth, and water content were enhanced in all tested conditions and both varieties, i.e., Faisal and Shiralee. Proteomic and gene expression analysis of the Zn NPs-treated plants revealed that the NPs have the ability to influence protein levels and gene transcription, which may explain the alteration of the morphological and physiological parameters. The selected genes expression analysis showed that genes related to photosynthetic pathways, SOD activity, defense, response to the biotic stress, and the structural constituents of the ribosomes. The findings of this study endorse the foliar applications of the Zn NPs to induce gene expression and alter the protein level of certain genes/proteins of the plant to increase biomass, RWC, and plant chlorophyll and carotenoid contents. Due to very limited literature, the role of Zn NP in the gene expression related to growth and production is not very cleared. Future studies are needed to be carried out to understand the molecular role of Zn NPs on plant physiological process.

## Data Availability Statement

The original contributions presented in the study are included in the article/supplementary material, further inquiries can be directed to the corresponding authors.

## Author Contributions

Sohail: writing, review, analysis, investigation, methods, and research. LS, EF, Y-DS, and BK: editing and review. All authors contributed to the article and approved the submitted version.

## Conflict of Interest

The authors declare that the research was conducted in the absence of any commercial or financial relationships that could be construed as a potential conflict of interest.

## Publisher’s Note

All claims expressed in this article are solely those of the authors and do not necessarily represent those of their affiliated organizations, or those of the publisher, the editors and the reviewers. Any product that may be evaluated in this article, or claim that may be made by its manufacturer, is not guaranteed or endorsed by the publisher.
